# Bovine Mastitis-Derived *Bacillus cereus* in Inner Mongolia: Strain Characterization, Virulence Factor Identification, and Pathogenicity Validation

**DOI:** 10.3390/vetsci12111057

**Published:** 2025-11-03

**Authors:** Chen Yu, Kollie Helena Vivian, Shuangyuan Fan, Xiaojiao He, Jingwen Zhao, Zhangping Yang, Kai Zhang, Tianle Xu

**Affiliations:** 1 Joint International Research Laboratory of Agriculture and Agri-Product Safety, Ministry of Education of China, Yangzhou University, Yangzhou 225009, China; 2 College of Animal Science and Technology, Yangzhou University, Yangzhou 225009, China; 3 Key Laboratory for Crop and Animal Integrated Farming of Ministry of Agriculture and Rural Affairs, Animal Husbandry Institute, Jiangsu Academy of Agricultural Sciences, Nanjing 210014, China

**Keywords:** bovine mastitis, *Bacillus cereus*, drug resistance, virulence factors

## Abstract

**Simple Summary:**

We conducted a study to investigate the role and characteristics of *Bacillus cereus* in bovine mastitis within dairy herds in Inner Mongolia, China. The objectives were to determine its prevalence, antimicrobial resistance profile, virulence gene carriage, and pathogenic potential. A total of 340 bacterial strains were isolated from clinical mastitis milk samples. *Bacillus cereus* was identified as the predominant pathogen. Antimicrobial susceptibility testing revealed significant resistance to tetracycline, but consistent susceptibility to gentamicin, amikacin, and roxithromycin. PCR assay confirmed that all *B. cereus* isolates carried key virulence genes. Mouse model challenges demonstrated that strains harboring a higher number of virulence genes induced more severe histopathological damage in liver and kidney tissues and a stronger inflammatory response. Our findings establish *Bacillus cereus* as a major etiological agent of mastitis in this region with a distinct resistance and virulence pattern. This study provides crucial epidemiological data to inform targeted mastitis control strategies and support animal health and food safety.

**Abstract:**

This study aimed to investigate the epidemiological characteristics and antimicrobial resistance patterns of *Bacillus cereus* (*B. cereus*) in bovine mastitis within Inner Mongolian dairy herds, with a focus on virulence gene distribution and their clinical implications. A cross-sectional epidemiological investigation was conducted across three large-scale dairy farms. A total of 340 bacterial strains were isolated from milk samples collected from 108 cows with clinical mastitis, all of which underwent comprehensive PCR testing. Antimicrobial susceptibility testing was performed using the Kirby–Bauer disk diffusion method against eight antibiotics. Virulence gene profiling was conducted for all *B. cereus* isolates, and murine challenge experiments were performed to assess virulence-factor-dependent pathogenicity. Bacteriological analysis identified *B. cereus* as the predominant pathogen (104 strains, 30.58%), followed by *Staphylococcus* spp. (74 strains, 21.76%). Antimicrobial susceptibility testing revealed high resistance to tetracycline (38.46%), cotrimoxazole (15.38%), and ciprofloxacin (7.69%), while complete sensitivity (100%) was observed for gentamicin, amikacin, and roxithromycin. Virulence gene profiling demonstrated universal presence of *nheA*, *nheB*, and *entFM* genes in all isolates, with *bh1D* detected in only 21.15% (22/104) of strains. Murine challenge experiments confirmed virulence-factor-dependent pathogenicity, with strains harboring nine virulence factors inducing significant upregulation of hepatic inflammatory markers (*p* < 0.05) and histopathological alterations in hepatic and renal tissues compared to strains with three virulence factors. Our findings highlight *B. cereus* as an emerging virulent pathogen in Inner Mongolian dairy herds, necessitating enhanced surveillance of virulence factors and antimicrobial stewardship in mastitis management. This study provides critical epidemiological data to inform clinical veterinary practices and targeted intervention strategies.

## 1. Introduction

Bovine mastitis is a common inflammation of the mammary gland that seriously affects the health of dairy cows and dairy production worldwide and even threatens public health [1]. Bovine mastitis remains one of the most perplexing and costly diseases in dairy cattle; thus, further research into its role and molecular mechanisms is needed [2]. Control of mastitis relies on effective strategies for dairy herds rather than identification or specific treatment of individual animals [3]. It is vital to identify the pathogenic microorganisms causing mastitis and the proportion of infected cows with a high somatic cell count (SCC) [4]. Bacteria such as *Staphylococcus aureus*, *Mycoplasma bovis*, *Corynebacterium bovis*, and *Streptococcus lactis* are prime pathogens of contagious agents [5]. In contrast, environmental mastitis is suggested to be associated with intramammary infections caused by microorganisms that originate primarily from the environment, such as *Escherichia coli* and *Klebsiella* spp. [6]. However, due to the emphasis placed on the management of dairy farming, the incidence of contagious mastitis has declined significantly in recent decades, whereas that of environmental mastitis has risen dramatically [7].

As early as 2008, data from the European Union showed that *Bacillus cereus* was a significant cause of food poisoning in humans and a significant cause of 1.4–12% of foodborne illnesses worldwide [8,9]. *B. cereus* is a Gram-positive bacterium that is aerobic or partially anaerobic; it can form spores in pastures and is widely found in bedding, sewage, air, and other environments [10]. Numerous studies have shown that *B. cereus* may cause mastitis in dairy cows through the environment [11]. Notably, *B. cereus* spores can germinate and grow to high levels in pasteurized milk and various dairy products, raising potential safety hazards for milk consumers [12].

Studies have shown that *B. cereus* causes gastrointestinal disorders in animals and humans [13], In China, foodborne illness outbreaks involving *B. cereus* are usually caused by dairy products [14], with the main symptoms of *B. cereus*-related food poisoning being diarrhea and vomiting [15]. Diarrhea is mainly caused by three enterotoxins belonging to the family of pore-forming toxins (PFTs) [16], including non-hemolytic enterotoxins (Nhe) [17], Hemolysin BL (Hbl) [18], and cytolysin K (CytK) [19]. Emetic syndrome is closely linked to a deadly toxin known as cereulide, which is synthesized by the non-ribosomal peptide synthetase (NRPS) encoded by the Ces Cereulide Synthetase (Ces) gene [20]. Low-dose cereulide exposure disrupts the intestinal barrier’s function and causes intestinal inflammation, which results from activation of the endoplasmic reticulum (ER) stress IRE1/XBP1/CHOP pathway, to induce cell apoptosis and inflammatory cytokine production. Regarding gut microbiota, cereulide decreases the abundances of Lactobacillus and Oscillospira. Furthermore, cereulide disrupts the metabolism of gut microbiota, which inhibit butyrate and tryptophan [21]. However, the correlations of various virulence genes carried by *B. cereus* and its toxicity remain unknown. In addition to enterotoxins, some *B. cereus* isolates are resistant to antimicrobial agents. Due to the widespread use of various antimicrobial drugs, the problem of bacterial resistance is becoming increasingly serious and poses a great threat to animal health [22]. Improving feeding efficiency and management are effective measures to prevent the occurrence of mastitis in dairy cows, but it cannot be completely eradicated. The use of antibiotics is still the main method of treatment in practice. Pathogenic microorganisms become resistant to drugs, leading to the enrichment of drug residues in dairy products and threatening food safety and health. In summary, it is necessary to study the resistance capacity, virulence factors, and virulence of the main pathogenic microorganisms causing mastitis in dairy cows. This study aims to investigate the dominant pathogenic groups of mastitis in dairy cows in Inner Mongolia. It also aims to assess the distribution of drug resistance and virulence genes from isolated *Bacillus cereus* and determine its pathogenicity by using a mouse model. The results may provide an experimental basis for the prevention and control of the epidemic spread of *Bacillus cereus* and help uncover its pathogenic severity regarding clinical mastitis in Inner Mongolia.

## 2. Materials and Methods

### 2.1. Isolation and Identification of Milk-Derived Bacteria

In this study, 108 milk samples of dairy cows with clinical mastitis from three farms (Farm A, B, and C) in Inner Mongolia were isolated and identified using a combination of bacterial culture and 16S rDNA sequencing method [23]. Farm A collected 52 samples, Farm B collected 48 samples, and Farm C collected 8 samples. Briefly, 80 µL of the milk sample was applied to the surface of blood agar using a glass rod and placed in a 37 °C incubator for 16 h. The external morphology of the bacterial colony was observed, and the parameters of size, shape, and color of the grown bacteria were recorded. The single colony was picked to grow in 3 mL of Luria–Bertani Broth, which was then placed in a 37 °C shaker for 16 h. The purification culture was carried out, and the DNA extraction of the pathogenic bacteria was carried out immediately after the completion of the culture. The identification of the 104 *B. cereus* isolates was performed using 16S rDNA sequencing combined with PCR-based confirmation. Briefly, genomic DNA was extracted using a standard bacterial DNA isolation kit (e.g., TIANGEN DNA Kit). The 16S rDNA gene region was amplified with universal primers (27F/1492R) under optimized PCR conditions. PCR conditions were 98 °C for 2 min, followed by 35 cycles at 98 °C for 10 s, 58 °C for 10 s, and 72 °C for 45 s with a final extension of 72 °C for 1 min. The 25 μL reaction mixture for PCR consisted of 1 μL each of forward and reverse primers, 0.5 μL of genomic DNA, 11.25 μL of Taq Master Mix and supplemented with sterile water up to 25 μL. After PCR amplification, the PCR products were detected using gel electrophoresis. 5 µL of all samples were pipetted into a 1% agarose gel for electrophoresis. After electrophoresis, a gel imaging system was used to observe the presence of bands, and the sample was sent to a sequencing company for sequencing. The sequencing results were subjected to BLAST comparison on the NCBI website, https://blast.ncbi.nlm.nih.gov/Blast.cgi (accessed on 15 February 2023), to determine the genus of the pathogenic bacteria.

### 2.2. Determination of Antimicrobial Susceptibility

In this experiment, 104 strains of *B. cereus* were subjected to drug sensitivity test and eight antibiotics were selected for use: erythromycin, tetracycline, ciprofloxacin, clindamycin, cotrimoxazole, gentamicin, roxithromycin, and amikacin [24]. The drug resistance phenotype of the bacteria was detected by paper diffusion method with reference to the CLSI 2021 guidelines for drug susceptibility testing, which categorized them as resistant (R), intermediary (I) and sensitive (S) (Figure S1) [25,26]. First, the culture medium was made, and the Mueller-Hinton Broth drug-sensitive agar was autoclaved after proportioning according to the instructions, then the bacterial solution was diluted, and the bacterial solution was diluted with saline at 1:10, and compared with the turbidimetric tubes of 0.5 McDonald’s units, and then the sterilized cotton swabs were dipped into the diluted bacterial suspension and evenly coated on the Mueller-Hinton agar, and then finally the drug-sensitive slices with the lettering side down were placed in the incubator at a constant temperature of 37 °C for incubation. When the incubation was completed, the bacterial growth around the drug-sensitive tablets was observed, and the diameter of the inhibition circle was measured by vernier calipers.

### 2.3. Detection of Virulence Genes

Firstly, the genomic DNA of *B. cereus* was extracted according to the instructions of the DNA extraction kit; then the DNA concentration was determined: the concentration of the extracted bacterial DNA was determined by a nucleic acid protein analyze; then the DNA concentration was diluted to 20 μg/mL and used as the template of the PCR reaction, which was stored at −20 °C for backup; finally, the PCR amplification was carried out for 11 virulence-related genes of *B. cereus* (*hblA*, *hblC*, *hblD*, *nheA*, *nheB*, *nheC*, *entFM*, *cytK*, *bceT*, *ces*, *EMl*) were amplified by PCR. The 25 μL reaction mixture for PCR consisted of 1 μL each of forward and reverse primers (Table S1), 2 μL of genomic DNA, 2 μL of deoxyribonucleoside triphosphates (dNTPs), 2.5 μL of 10 × Ex Taq Buffer (Mg^2+^ Plus), 0.3 μL of Ex Taq (5 U·μL^−1^) and supplemented with sterile water up to 25 μL. For the selected isolate the purified PCR products of the target genes in the selected isolates were sequenced and analyzed at NCBI.

### 2.4. Construction of Mouse Mastitis Model

For the experiments, 6–8-week-old mice were obtained from the Center of Comparative Medicine at Yangzhou University. All experimental procedures and care of experimental animals in the study were followed according to the Manual for the Care and Use of Laboratory Animals published by the National Institutes of Health. The mice were randomly divided into 3 groups (n = 6/group): the **control group** (**NC**), the group injected with the least virulence genes of *B.cereus* was **LVB** (***B. cereus*** **IM01**, ***nheA***, ***nheB***, ***entFM*** **expressed**), and the group injected with the most virulence genes of *B.cereus* was **MVB** (***B. cereus*** **IM21**, ***nheA***, ***nheB***, ***entFM***, ***nheC***, ***hblA***, ***hblC***, ***hblD***, ***cytK***, ***bceT*** **expressed**). The 5 × 10^8^ CFU/mL attacking bacterial solution was prepared and injected into the ducts of the fourth mammary gland area for 24 h, and the morbidity and mortality were counted, and the dead and abnormal mice were promptly dissected. After killing the mice, the livers and kidneys were aseptically collected for hematoxylin-eosin (H&E) staining microscopy to observe the tissue lesions. At the same time, serum was collected for the determination of biochemical parameters.

### 2.5. Histological Analysis

Pieces of tissue from mice were fixed in buffered formalin phosphate (10%), followed by paraffin-embedding, sectioned, and stained with hematoxylin and eosin, and a phase-contrast microscope was used for capturing (Nikon, Tokyo, Japan). Histopathological changes in the murine tissues were evaluated according to the sum of the infiltration of inflammatory cells, the vascular endothelium injury, and the disorder of sinuses and lobules in the liver and kidney. Each histological characteristic was evaluated on a scale of 0–5.

### 2.6. Determination of Biochemical and Oxidative Stress Parameters in Serum and Liver Tissue

The level of ALT, AST, LDH, and ALP of serum and GSH-Px, SOD, and MDA content of liver tissues were determined using commercial kits from Nanjing Jianjian Biological Company by the instructions.

### 2.7. RNA Extraction and Quantitative PCR Analysis

Total RNA from mice hepatic tissues was isolated with the RNA Isolater Total RNA Extraction Reagent (R401-01, Vazyme, Nanjing, China) according to the instructions of manufacturer. cDNA was synthesized by applying HiScript III RT SuperMix (R323-01, Vazyme, Nanjing, China) thereafter purified with purification kit (Axygen, Tewksbury, MA, USA). In brief, qRT-PCR was performed by using AceQ Universal SYBR qPCR Master Mix (Vazyme, Nanjing, China) on an ABI QuantStudio System (Applied Biosystems, Foster City, CA, USA). The PCR program was designed as follows: an initial denaturation at 95 °C for 30 s, followed by a cycling stage consisting of denaturation at 95 °C for 5 s, annealing at 60 °C for 30 s, and 40 cycles. Subsequently, the extension was performed at 95 °C for 15 s and 60 °C for 60 s, followed by a final extension at 90 °C for 15 s to generate amplification curves. The housekeeping gene, GAPDH, was used as an internal reference. Each RT-qPCR was set up in triplicates, and the experiment was repeated three times [27]. The target gene expression data were normalized using the geometric mean of the internal control genes. Relative quantification was performed using the 2^−ΔΔCt^ method [28].

### 2.8. Western-Blotting Analysis

Western blot was performed using protocols described previously [29]. For Western blot analysis, 6 individual samples per group (n = 6 per group, total 3 groups) were collected. To reduce sample volume while maintaining statistical power, samples from each group were pooled in pairs, resulting in 3 pooled biological replicates per group. All samples from experimental mice were included in the analysis. In brief, equal amounts of protein isolated from hepatic tissues by RIPA lysis buffer (Beyotime, Shanghai, China) were separated on 4% to 20% SDS polyacrylamide gels. Protein samples were transferred onto nitrocellulose membranes (Millipore, Billerica, MA, USA) and probed with primary antibodies overnight at 4 °C. Following six washes, the membranes were incubated with horseradish peroxidase (HRP)-conjugated secondary antibodies. To account for variations in protein transfer efficiency between blots, GAPDH levels were used for normalization. Band intensities were quantified using Bio-Rad imaging software V5.2.1(Bio-Rad, Hercules, CA, USA) by measuring the gray values of each target protein. Primary antibodies for *p-P65*, *p65*, *TNF-α*, *TLR4*, *IL-6*, and *IL-8* were purchased from Cell Signaling Technology (Danvers, MA, USA; #3033, #8242, #6945, #14358, #12153, #94407), and were diluted 1:1000 for incubation.

### 2.9. Statistics

Multiple comparisons among groups were carried out by one-way analysis of variance (ANOVA) with Tukey’s post hoc test. Differences between two groups were assessed by a two-tailed Student’s *t*-test, with *p* < 0.05 indicating significant differences, and the results were expressed as the means ± SEM. Figures were drawn using GraphPad Prism 10.4.0 (GraphPad Software, Inc., San Diego, CA, USA).

## 3. Results

### 3.1. Isolation of Bacterial from Bovine Clinical Mastitis

A total of 340 strains of bacteria were detected in 108 clinical mastitis milk samples. Table 1 shows that only two of the milk samples from farm B did not contain bacteria, while bacteria were detected in 98.14% of milk samples from the three farms. In total, 12 species with 340 strains of bacteria were isolated in this study (Table 2). Among the isolated strains, *Bacillus cereus* had the highest detection rate of 30.58% (104/340), followed by *Staphylococcus aureus* at 21.76% (74/340); *Enterococcus faecalis*, *Arthrobacter*, and *Actinobacillus* had the lowest detection rate of 0.59% (2/340). The distribution of detected bacteria in each farm is also shown in Table 2. *B. cereus* and *Staphylococcus aureus* were detected in all three farms, with *B. cereus* being the most abundant in farm A and the least in farm C. The distribution of *S. aureus* is consistent with that of *B. cereus*, both being the most abundant in farm A and the least in farm C. *Rhizobium* only appeared in farms A and B at a rate of 7.65%; *Streptococcus agalactiae* was only found in farm A; and *Copperhead* and *Arthrobacter* were only found in farm B at rates of 0.17% and 0.59%, respectively. *Enterococcus faecalis*, *Actinobacillus*, and *Pseudomonas* were found in farm A with detection rates of 0.59%, 0.59%, and 2.35%, respectively.

### 3.2. Phenotypic Drug Susceptibility of B. cereus Isolated from Bovines with Clinical Mastitis

**Figure 1:**
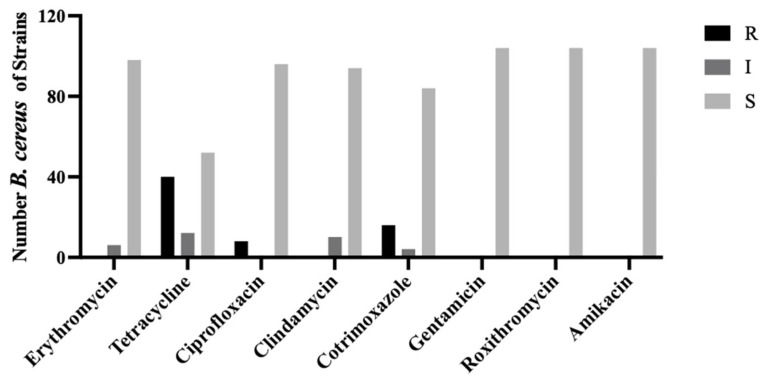
Susceptibility of *B. cereus* to different antibiotics. Using 4 kinds of 8 antibiotics to analyze the susceptibility of 104 *B. cereus*. Black, dark gray, and light gray represent for drug resistance levels with resistant (R), intermediate (I), and sensitive (S).

Figure 1 shows that 104 strains of *Bacillus cereus* were tested for resistance to eight antibiotics. The strains showed the strongest resistance to tetracycline at 38.46%, followed by cotrimoxazole and ciprofloxacin with resistance rates of 15.38% and 7.69%, respectively. Additionally, there was strong susceptibility to erythromycin and clindamycin. All *B. cereus* strains were sensitive to gentamicin, amikacin, and roxithromycin.

### 3.3. Carriage of Virulence Genes in B. cereus

The main virulence factors carried by 104 strains of *B. cereus* from milk samples were analyzed, and the frequency of detection of each virulence gene is shown in Table 3. All *B. cereus* strains carried *nheA*, *nheB*, and *entFM* genes, with only 22 strains carrying the *hb1D* gene. The distribution of virulence genes of the strains is shown in Table 4; 99.90% of *B. cereus* strains carried four or more virulence factors. Among them, 16 strains carried five genes (*nheA*, *nheB*, *entFM*, *nheC*, and *bceT*), 4 strains simultaneously expressed nine virulence factors, and 102 strains co-expressed *nheA*, *nheB*, and *nheC*.

### 3.4. B. cereus Induces Histopathological Damage in the Liver and Kidney and Inflammatory Gene Expression in the Liver

**Figure 2:**
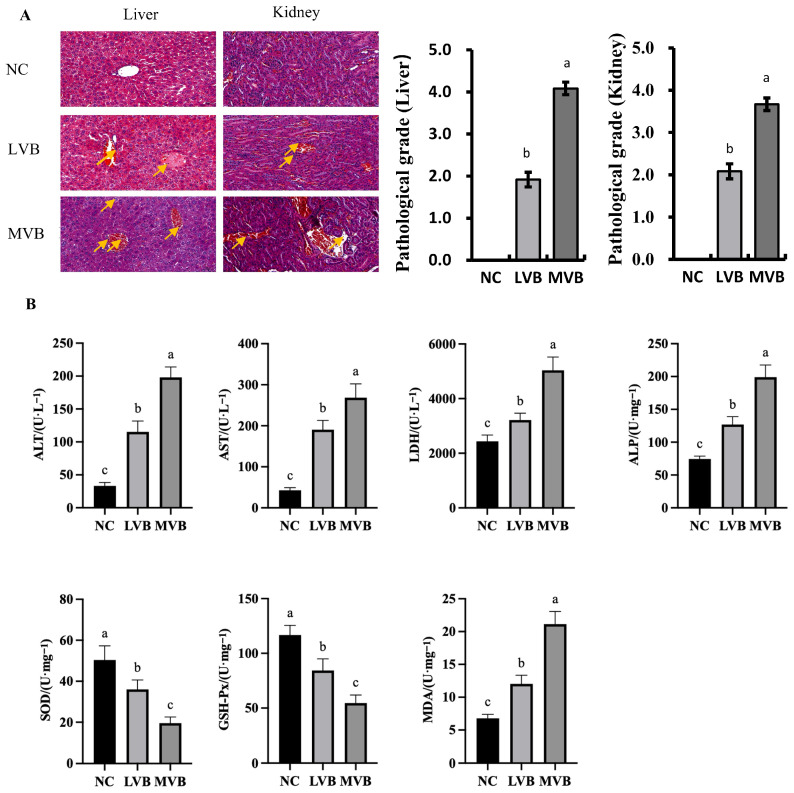
Phenotypic and biochemical parameters detected in mice infected with *B. cereus*. (**A**) Histopathological analysis and evaluation of the inflammatory score of two strains of *B. cereus* with the least (LVB) or most (MVB) number of virulence genes carried on the liver and kidney tissue of infected mice. Inflammatory scores were calculated according to the sum of the vascular endothelium injury and the disorder of sinuses and lobules in the liver and kidney, and the number of infiltration cells. Arrows indicate typical inflammatory cells infiltrated. (**B**) Effects of two strains of *B. cereus* on ALT, AST, LDH, ALP, SOD, GSH-Px, and MDA in mice. All data are presented as the mean value ± Standard Error of the Mean (SEM), and n = 6 in each group. The letters in superscript indicate that the difference between groups was significant (*p* < α, where α = 0.05).

Two strains of *B. cereus* carry different numbers of virulence genes (one isolate carried the least virulence genes, while another carried the most) and induced inflammatory responses in the livers and kidneys of mice. Histopathological changes in the murine tissues were evaluated according to their injury degree score, including the infiltration of inflammatory cells (as the arrows shown), the vascular endothelium injury, and the disorder of sinuses and lobules in the liver and kidney. Each histological characteristic was evaluated on a scale of 0–5, as described previously [30]. According to Figure 2A, both infected groups showed infiltration of inflammatory cells in liver and kidney tissues, congestion of the tissue interstitial space, and obvious destruction of tissues, demonstrating the toxic effects of *B. cereus*. The destruction of the livers and kidneys was significantly enhanced in the MVB group compared to the LVB group.

### 3.5. Levels of Biochemical Parameters in Plasma of Mice Induced by B. cereus

Data on the biochemical parameters are shown in Figure 2B. The LVB and MVB groups were treated by different strains of *B. cereus*, which led to a significant increase in hepatic inflammatory responses in the mice with increased carriage of virulence factors compared to the control group. The plasma concentration of Alanine aminotransferase (ALT), Aspartate aminotransferase (AST), Lactate dehydrogenase (LDH) and Alkaline phosphatase (ALP) were significantly higher in both treatment groups than in the control group. Moreover, plasma in the MVB group demonstrated higher secretion of ALT, AST, LDH, and ALP than that in the LVB group. Among oxidative stress factors, Superoxide dismutase (SOD) and Glutathione peroxidase (GSH-Px) were significantly lower in both treatment groups (LVB and MVB group) than in the control group. Additionally, the concentrations of SOD and GSH-Px significantly decreased in the MVB group compared to the LVB group. The concentration of Malondialdehyde (MDA) in LVB and MVB plasma were significantly upregulated compared to the control group. The results of these indexes indicate that the livers of the treated mice were severely damaged, worsening with the increased carriage of virulence factors.

### 3.6. The Inducible Effect of B. cereus on the Expression of Inflammatory Genens and Proteins

**Figure 3:**
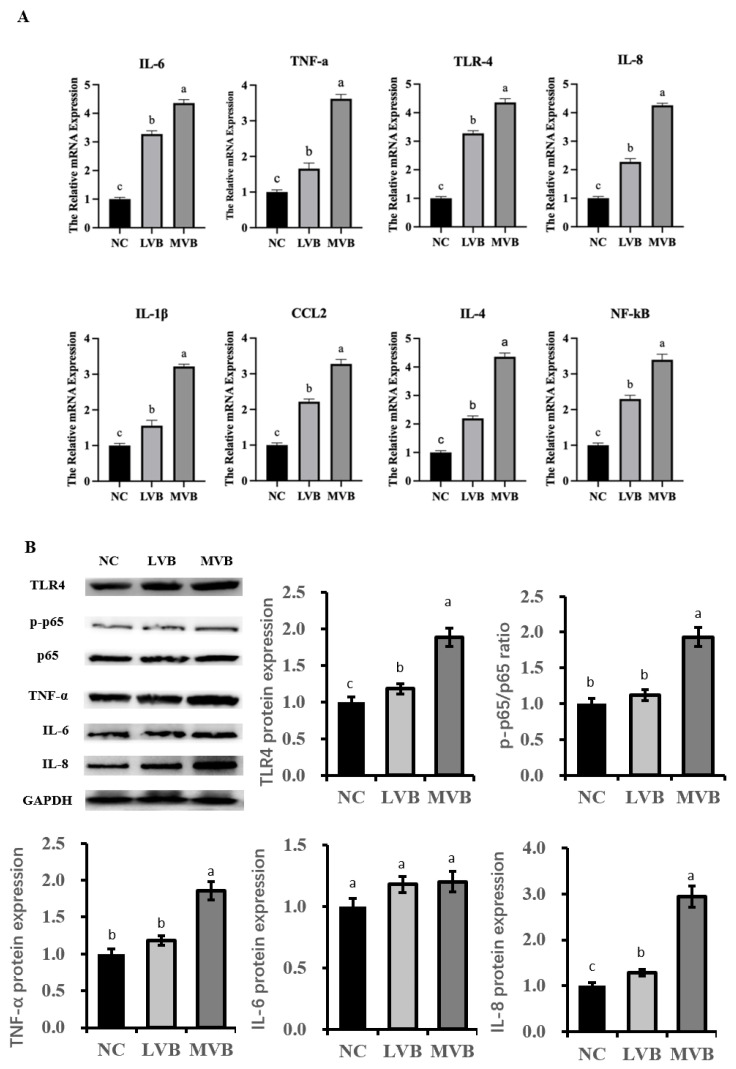
Effect of two strains of *B*. *cereus* with least (LVB) or most (MVB) numbers of carried virulence genes on inflammatory gene (**A**) and protein (**B**) expression in the liver of mice. All data are presented as the mean value ± Standard Error of the Mean (SEM), and n = 6 in each group. The letters in superscript indicate that the difference between groups was significant (*p* < α, where α = 0.05).

The two selected strains of *B. cereus* carrying different numbers of virulence genes induced significantly different expressions of inflammatory genes in mouse liver. Figure 3A shows that the relative mRNA expression of pro-inflammatory factors was significantly higher in both *B. cereus*-treated groups than in the control group. The expression of inflammatory genes on mouse liver in the MVB group (involving mice challenged by *B. cereus* containing nine virulence genes) was significantly stronger than that in the LVB group (involving mice attacked by *B. cereus* containing one virulence gene).

The expression of proteins related to inflammatory responses, namely *TLR4*, *p65*, *TNF-α*, *IL-6*, and *IL-8*, was determined to uncover the effects of *B. cereus* infection. Figure 3B shows that the expression of TLR4 in both the LVB and MVB groups was significantly upregulated compared to the control group (*p* < 0.05). Moreover, MVB had a higher abundance of *TLR4* protein than the LVB group. As the active subunit of NF-κB, the ratio of phosphorylated p65 to total p65 was significantly increased in the MVB group compared to the NC and LVB groups (*p* < 0.05). Similarly, compared to the NC and LVG groups, the expression of *TNF-α* and *IL-8* in the MVB group was markedly upregulated (*p* < 0.05). However, LVB challenge did not affect the expression of *TNF-α* and *IL-8* compared to the control group. Notably, the abundance of *IL-6* in either the LVB or MVB group did not differ from that in the control group.

## 4. Discussion

In this study, 108 milk samples collected from clinical mastitis cases on three dairy farms in Inner Mongolia were subjected to pathogenic bacteria isolation and characterization. The results demonstrate that the total detection rate of bacteria was 98.14%, confirming that bacterial infections remain the primary cause of mastitis in dairy cows, which aligns with previous studies [31]. *B. cereus* is the pathogenic bacteria species that is increasingly identified in cases of bovine mastitis and in dairy products [32]. The multi-bacterium infection rate was 81%, suggesting that mastitis treatment is complicated by the diversity of pathogens in this region. Furthermore, the detection rate of Gram-positive bacteria was higher than that of Gram-negative bacteria, with *B. cereus* exhibiting the highest detection rate. This finding is markedly different from the prevalent strains of bovine mastitis reported in some areas of Jiangsu Province [33,34], highlighting significant regional variations in mastitis-causing strains across China. *B. cereus*, an important conditional pathogen for mastitis, was detected in all three farms. Previous research has shown that *B. cereus* is widely distributed in the north of China, further supporting its role as a primary causative agent of mastitis in dairy cows in this region [35]. Thus, it is essential to not only timely and effectively eliminate and control common mastitis pathogens on dairy farms but also actively prevent *B. cereus* and other opportunistic pathogenic bacteria to mitigate the occurrence and spread of mastitis in this region. The pathogenic bacteria isolated in the current study encompass both contagious and environmental pathogens, indicating that environmental factors contribute to clinical mastitis infections and cross-infection among cattle. Therefore, farms should maintain dry bedding, remove manure promptly, and regularly disinfect the environment and milking equipment to block the growth of pathogenic bacteria and reduce the incidence of mastitis in dairy cows.

Previous studies have shown that *B. cereus* is one of the pathogens responsible for mastitis in dairy cows. The current study demonstrated that *B. cereus*, the primary pathogen of mastitis in this specific region of Inner Mongolia, was highly resistant to tetracycline, cotrimoxazole, and ciprofloxacin but sensitive to clindamycin, erythromycin, gentamicin, roxithromycin, and amikacin. *B. cereus* isolated from food sources was found to be resistant to cotrimoxazole, which is consistent with the findings of this study [36]. Additionally, this study revealed that *B. cereus* is highly resistant to ciprofloxacin, which contrasts with the results of previous studies [37]. This discrepancy might be attributed to the fact that the *B. cereus* in this experiment was isolated from cows suffering from mastitis that were treated with medication that may have influenced antibiotic resistance [38]. Compared with the findings of Chang et al., *B. cereus* showed resistance to non-β-lactam antibiotics, and the degree of resistance varied slightly among different antibiotics, likely due to differences in *B. cereus* strains and specific drugs used [39]. Notably, while *B. cereus* has gained significant attention as a probiotic due to its beneficial effects, it must also be recognized as a conditional pathogen [40]. Under certain conditions, it can cause serious diseases in animals. The presence of drug-resistant strains of *B. cereus* identified in the current study shows that it poses a challenge to the dairy industry and is a significant threat to public health and safety.

Virulence factors are significant in the establishment of infection and survival of *B. cereus* in the host organism, and the pathogenicity of *B. cereus* is directly related to the virulence genes it carries. The *B. cereus* strain has been widely utilized as a probiotic for humans, food-producing animals, plants, and environmental remediation. Paradoxically, *B. cereus* also functions as a significant opportunistic foodborne pathogen, implicated in both gastrointestinal and extra-gastrointestinal syndromes [41]. In this study, we analyzed the primary virulence factors carried by 104 strains of *B. cereus* isolated from mastitis cases. The three subunit proteins of the *non-hemolytic enterotoxin* (*Nhe*) are encoded by *nheA*, *nheB*, and *nheC*. Among these, *NheB* plays a key role in the virulence of *Nhe*, whereas excess *NheC* inhibits the virulence of *Nhe*. However, these three subunits exist and are expressed simultaneously in cases of maximum toxicity. The *nheB* gene was detected in all 52 strains of *B. cereus* in this study, and 98% of the strains simultaneously carried the *nheA*, *nheB*, and *nheC* genes. Additionally, 32 strains of *B. cereus* carried the *hbl* gene, which binds to the cell membrane sequentially through its three subunits before disrupting the cell membrane and causing cell lysis. Thus, *Hbl* can only be produced by *B. cereus* that contains and expresses the three virulence genes *hblA*, *hblC*, and *hblD*. Five strains of *B. cereus* were found to carry all three virulence genes of both *nhe* and *hbl*, and all strains carried *entFM* genes in addition to the *nhe* and *hbl* genes. Furthermore, 38 strains carried the *bceT* gene, and 32 strains carried the *cytK* gene. The high prevalence of the *nhe*, *entFM*, *hbl*, *bceT*, and *cytK* genes indicate that they are the primary virulence factors of foodborne *B. cereus* in China; this is largely consistent with the results of previous studies [42]. This study also aligns with the results of Cui et al., who suggested that the presence of virulence genes in *B. cereus* is not an isolated phenomenon but a widespread occurrence [43]. With the extensive use of feed additives and the increasing adoption of biopesticides, *B. cereus* may pose potential risks to human health and food safety, particularly since *B. cereus* virulence-producing plasmids are capable of horizontal gene transfer. Additionally, the fact that many *B. cereus* spores carry enterotoxin genes further highlights the potential hazards associated with the use of biological products such as *B. cereus*. Therefore, it is crucial to maintain a high level of vigilance and conduct more in-depth safety assessments to ensure the safe and rational use of *B. cereus* in production and daily life [44].

Mouse models are easy to use and cost-effective. Wang et al. showed that the pathological changes in the mammary gland tissues of mice are similar to those observed in dairy cows with mastitis [45]. The ICR mouse used in this experiment is ideal for constructing a dairy cow mastitis model. Both groups capable of inducing acute mastitis were evaluated based on histopathological changes, inflammatory cytokine production, and the extent of liver and kidney tissue damage. We observed that the liver and kidney tissues in both the LVB and MVB groups exhibited inflammatory cell infiltration, interstitial congestion, and significant structural damage. Additionally, the expression of inflammatory genes in the liver was significantly upregulated in both the LVB and MVB groups compared to the control group, and hematological parameters were also significantly altered. Elevated serum levels of *TNF-α* are known to induce neutrophil activation and migration, leading to mammary epithelial apoptosis in both bovine and human patients with acute clinical mastitis [46,47,48]. Moreover, inflammatory cytokines can enhance multiple functions of inflammatory cells, including cell adhesion, cell surface receptor expression, release of lysosomal components, and free radical production [49]. In the present study, the levels of pro-inflammatory cytokines *IL-1β* and *IL-6* in the mammary glands of mice administered virulence factors were significantly elevated compared to the control group, indicating that cytokines play a critical role in mediating mammary tissue damage in mice. We further analyzed the pathogenic potential of virulence factors in a mouse mastitis model by directly injecting *B. cereus* suspensions containing varying numbers of virulence factors. The results demonstrated that *B. cereus* strains containing different levels of virulence factors induced varying degrees of tissue damage, confirming that the virulence of *B. cereus* is directly influenced by the virulence factors it carries.

## 5. Conclusions

Collectively, this investigation establishes *B. cereus* as the predominant etiological strain of mastitis across the Inner Mongolian dairy farms sampled, demonstrating distinct resistance and virulence gene profiles. Tetracycline resistance was observed in 38.46% of the *B. cereus* strains, constituting the highest resistance rate. Universal carriage of *nheA*, *nheB*, and *entFM* virulence determinants was identified across all isolates, with murine challenge experiments confirming virulence-gene-load-dependent pathogenicity. Strains harboring more virulence gene clusters demonstrated strengthened pathogenic potential, directly correlating with the severity of host tissue damage. These findings provide critical epidemiological evidence for developing region-specific mastitis control strategies in Inner Mongolia and emphasize the need to implement tetracycline stewardship programs to mitigate antimicrobial resistance proliferation in dairy production systems.

## Figures and Tables

**Table 1:** Detection and distribution analysis of pathogenic bacteria in clinical mastitis.

Items	Farm A		Farm B		Farm C		Total	
	Bacteria	None	Bacteria	None	Bacteria	None	Bacteria	None
Milk Samples (No.)	52	0	46	2	8	0	106	2
Rate (%)	48.15	0	42.59	1.86	7.4	0	98.14	1.86

**Table 2:** Detection and distribution analysis of pathogenic bacteria in clinical mastitis.

Name of Bacteria	Number of Detections	Detection Rate	Farm A	Farm B	Farm C
*Bacillus cereus*	104	30.58%	57	39	8
*Staphylococcus aureus*	74	21.76%	38	30	6
*Bacillus subtilis*	26	7.65%	12	10	4
*Rhizobium*	26	7.65%	24	2	0
*Bacillus sphaericus*	14	4.12%	7	6	1
*Bacillus pusillus*	12	3.54%	5	7	0
*Pseudomonas*	8	2.35%	8	0	0
*Streptococcus agalactiae*	4	1.17%	4	0	0
*Copperhead*	4	1.17%	0	4	0
*Enterococcus faecalis*	2	0.59%	2	0	0
*Arthrobacter*	2	0.59%	0	2	0
*Actinobacillus*	2	0.59%	2	0	0
Others	62	18.24%	38	16	8
Total	340	100%	197	116	27

**Table 3:** Different types of virulence factors carried by *B. cereus.*

Carrying Virulence Genes	Number of *B. cereus* Strains	Detection Rate
*nheA*	104	100%
*nheB*	104	100%
*entFM*	104	100%
*nheC*	102	98.1%
*cytK*	32	30.8%
*hblC*	42	40.4%
*hblA*	38	36.5%
*hblD*	22	21.2%
*bceT*	38	36.5%
*ces*	26	25%
*EM1*	26	25%

**Table 4:** Profile of virulence factors carried by *B. cereus.*

Carrying Virulence Genes	Amount of *B. cereus* Strains
*nheA*, *nheB*, *entFM*	2
*nheA*, *nheB*, *entFM*, *nheC*	10
*nheA*, *nheB*, *entFM*, *nheC*, *hblA*	4
*nheA*, *nheB*, *entFM*, *nheC*, *hblC*	8
*nheA*, *nheB*, *entFM*, *nheC*, *cytK*	6
*nheA*, *nheB*, *entFM*, *nheC*, *bceT*	16
*nheA*, *nheB*, *entFM*, *nheC*, *cytK*, *hblC*	2
*nheA*, *nheB*, *entFM*, *nheC*, *hblA*, *hblC*	2
*nheA*, *nheB*, *entFM*, *nheC*, *hblC*, *hblD*	2
*nheA*, *nheB*, *entFM*, *nheC*, *hblC*, *cytK*	4
*nheA*, *nheB*, *entFM*, *nheC*, *hblA*, *cytK*, *bceT*	6
*nheA*, *nheB*, *entFM*, *nheC*, *hblA*, *ces*, *EM1*	8
*nheA*, *nheB*, *entFM*, *nheC*, *bceT*, *ces*, *EM1*	6
*nheA*, *nheB*, *entFM*, *nheC*, *hblA*, *hblC*, *hblD*	2
*nheA*, *nheB*, *entFM*, *nheC*, *hblA*, *hblC*, *cytK*	2
*nheA*, *nheB*, *entFM*, *nheC*, *hblC*, *hblD*, *cytK*	2
*nheA*, *nheB*, *entFM*, *nheC*, *hblA*, *bceT*, *ces*, *EM1*	4
*nheA*, *nheB*, *entFM*, *nheC*, *hblC*, *hblD*, *ces*, *EM1*	8
*nheA*, *nheB*, *entFM*, *nheC*, *hblA*, *hblC*, *cytK*, *bceT*	2
*nheA*, *nheB*, *entFM*, *nheC*, *hblA*, *hblC*, *hblD*, *cytK*	4
*nheA*, *nheB*, *entFM*, *nheC*, *hblA*, *hblC*, *hblD*, *cytK*, *bceT*	4

## Data Availability

The raw data supporting the conclusions of this article will be made available by the authors on request.

## Supplementary Materials

The following supporting information can be downloaded at: https://www.mdpi.com/article/10.3390/vetsci12111057/s1, Figure S1: Typical figures of resistant (R), intermediary (I), and sensitive (S); Table S1: Primers used for detection of virulence genes in *B. cereus*.
