# Supplementary material for: Bovine Mastitis-Derived Bacillus cereus in Inner Mongolia: Strain Characterization, Virulence Factor Identification, and Pathogenicity Validation
Source: Vet Sci. 2025 Nov 3;12(11):1057. doi: 10.3390/vetsci12111057 (PMC12656856; doi:10.3390/vetsci12111057)

Table S1 Primers used for detection of virulence genes in *B. cereus*

Virulence gene		Primer sequence (5'-3')	Annealing temperature /℃	Amplicons length /bp
hblA	hblA-F<br>hblA-R	AAGCAATGGAATACAATGGG<br>AGAATCTAAATCATGCCACTGC	56	1 154
hblC	hblC-F<br>hblC-R	GATACTCAATGTGGCAACTGC<br>TTGAGACTGCTCGTCTAGTTG	58	740
hblD	hblD-F<br>hblD-R	ACCGGTAACACTATTCATGC<br>GAGTCCATATGCTTAGATGC	58	829
nheA	nheA-F<br>nheA-R	GTTAGGATCACAATCACCGC<br>ACGAATGTAATTTGAGTCGC	56	755
nheB	nheB-F<br>nheB-R	TTTAGTGGATCTGTACGC<br>TTAATGTTCGTTAATCCTGC	54	743
nheC	nheC-F<br>nheC-R	TGGATTCCAAGATGTAACG<br>ATTACGACTTCTGCTTGTGC	58	683
entFM	entFM-F<br>entFM-R	AAAGAAATTAATGGACAAACTCAAACTCA<br>GTATGTAGCTGGGCCTGTACGT	60	596
cytK	cytK-F<br>cytK-R	GTAACTTTCATTGATGATCC<br>GAATACATAAATAATTGGTTTCC	48	505
bceT	bceT-F<br>bceT-R	TTACATTACCAGGACGTGCTT<br>TGTTTGTGATTGTAATTCAGG	52	428
ces	ces-F<br>ces-R	GGTGACACATTATCATATAAGGTG<br>GTAAGCGAACCTGTCTGTAACAACA	58	1271
EMl	EMl-F<br>EMl-R	GACAAGAGAAATTTCTACGAGCAAGTACAAT<br>GCAGCCTTCCAATTACTCCTTCTGCCACAGT	60	635

Figure S1 Typical figures of resistant (R), intermediary (I), and sensitive (S)

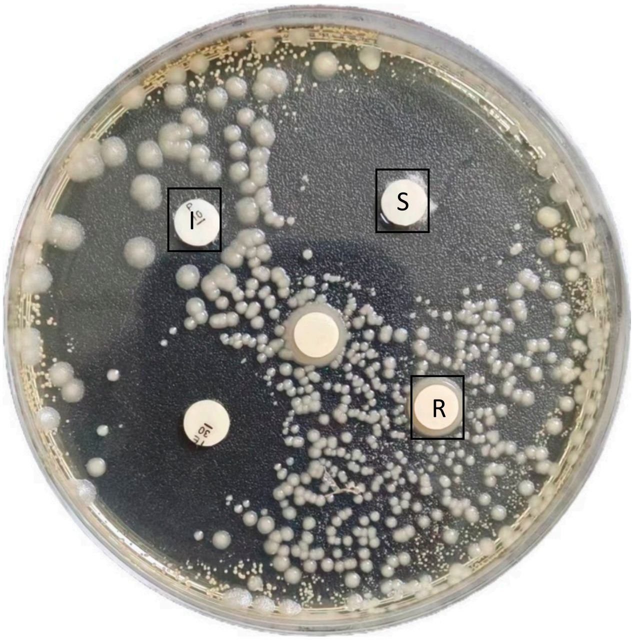

Supplement: Supplementary file 1 [file vetsci-12-01057-s001.zip › vetsci-3927829-supplementary.pdf]
